# Feasibility and Usability of Home-Based Visual Evoked Potential Electroencephalogram in Neurological Telemonitoring Research

**DOI:** 10.34133/csbj.0081

**Published:** 2026-05-15

**Authors:** Annemijn S.J.C. Oosterlee, Jet A.C. Jongenelen, Merel J. Kroon, Jamie L. Duijvelshoff, Paul Koster, Mark Ruit, Gisela M. Terwindt

**Affiliations:** ^1^ Department of Neurology, Leiden University Medical Center, Leiden, The Netherlands.; ^2^ Department of Biomechanical Engineering, Delft University of Technology, Delft, The Netherlands.; ^3^ Clinical Science Systems, Leiden, The Netherlands.

## Abstract

**Background:** Telemedicine offers promising tools for migraine care and research. A home-based visual evoked potential electroencephalogram (VEP-EEG) system was developed to help predict migraine attacks. **Objective:** To evaluate the usability and user-friendliness of a home-based VEP-EEG setup for research purposes. **Methods:** One group of 20 participants evaluated the first VEP-EEG setup. Another group of 20 participants evaluated the second setup. Setup 2 was modified based on feedback from group 1. Each participant completed 2 measurement days on separate occasions. Usability was assessed using the System Usability Scale (SUS), task completion time, number of successfully connected electrodes, and overall user experience. **Results:** Both setups scored high on usability: Setup 1 had a median SUS of 91.2 [interquartile range 86.2 to 95.0], setup 2 scored 81.2 [70.6 to 90.6]. Group 1 showed a significant reduction in task time between sessions (−3.3 min, *P* < 0.001); no significant change was observed for group 2. Successful electrode connection rates were similar (median 6/7). Overall experience was rated highly by participants (median 4/5). **Conclusion:** Home-based VEP-EEG telemonitoring is feasible and user-friendly. **Significance:** These results support its use in remote research and broader applications in studying paroxysmal brain disorders; however, further studies are needed to validate EEG signal quality.

## Highlights

• A home-based visual evoked potential EEG (VEP-EEG) system was developed to help predict migraine attacks.
• Complex telemonitoring in a research setting has been shown to be feasible.
• The improved VEP-EEG setup, with direct feedback on electrode connection, demonstrates good usability and user-friendliness for home-based research.

## Graphical Abstract

**Figure:**
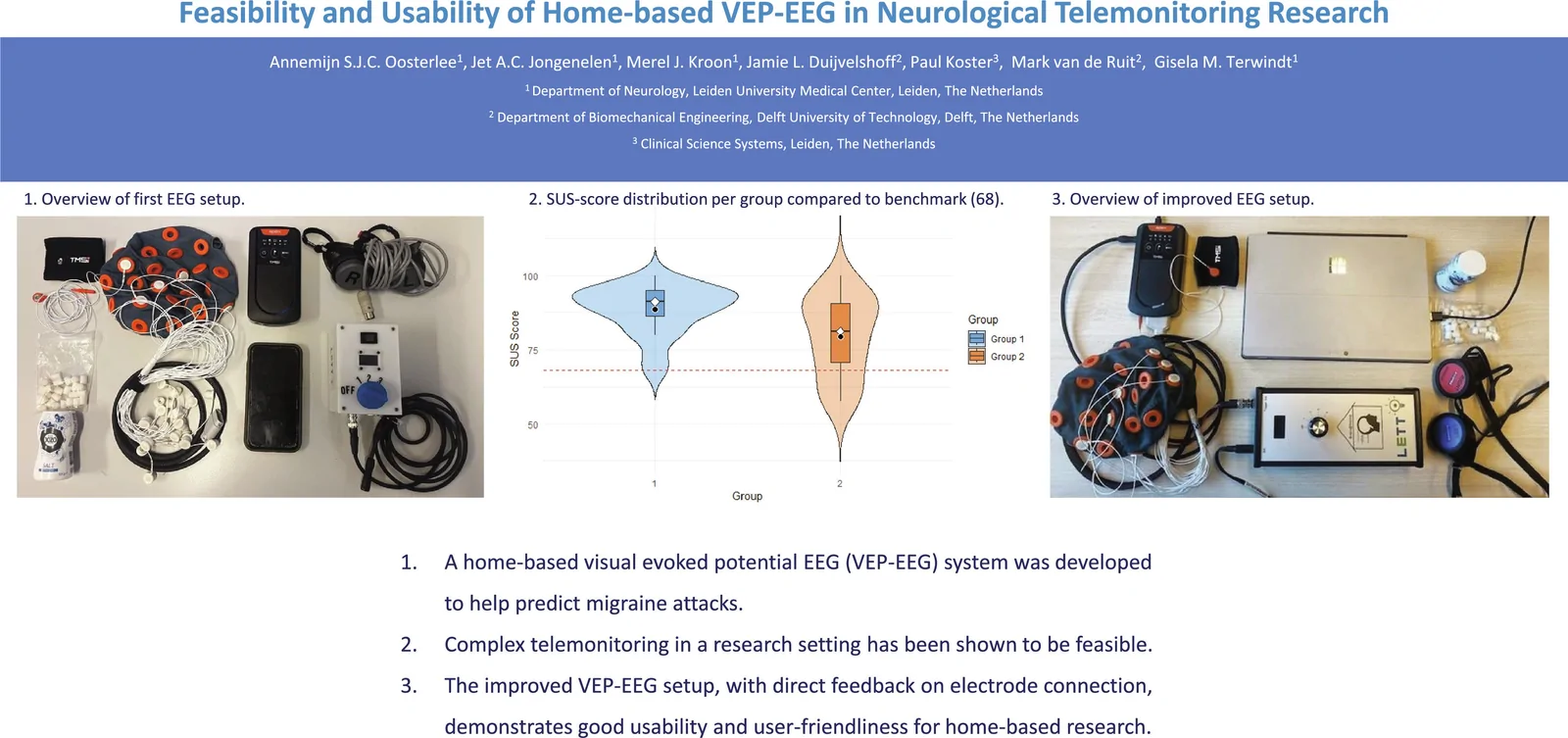

## Introduction

Telemedicine and e-health solutions are increasingly recognized as effective options in migraine care. Teleconsultations provide a viable alternative to traditional in-person visits while maintaining comparable quality of care [1]. Telemedicine approaches are particularly suitable for individuals with migraine, as treatment and symptom monitoring can be facilitated through the use of electronic diaries. These tools not only enhance treatment reliability but also contribute to improved diagnostic accuracy [2]. Prior research has demonstrated that electronic diaries help reduce recall bias [2] and have even supported the development of refined definitions for migraine-related clinical end points, such as migraine days [3].

Given the established value of telehealth and digital tools in migraine treatment and diagnostics, their role in migraine research warrants further exploration. This study focuses on evaluating the usability and user-friendliness of a system enabling self-administered electroencephalogram (EEG) measurements by participants in response to visual evoked potential (VEP) stimulation in their home environment. Insights from this evaluation will inform the optimization of a subsequent project aiming to monitor cortical excitability changes during the preictal phase of migraine attacks, with the goal of identifying early markers of impending attacks. Previous research has indicated that EEG responses to visual chirp stimulation (VEP-EEG) may reflect altered cortical responsivity during the preictal phase [4]. To validate these findings on a larger scale, the implementation of home-based measurements using electronic tools is essential. Another study in patients with epilepsy found a wearable EEG system, electronic diary, and smartwatch highly acceptable and usable for daily home monitoring [5]. However, for migraine patients, a different approach is needed, as continuous EEG registration is not the goal, but rather a daily snapshot of their cortical excitability state, that can serve as an early warning tool for upcoming attacks. This means that a shorter recording time would be sufficient, in contrast with epilepsy patients.

A setup was developed to enable participants to perform self-administered VEP-EEG measurements at home, utilizing e-health tools and a cap equipped with water-based electrodes. The signal quality of water-based electrodes has previously been found to be comparable to the gold standard of traditional gel-based systems. Importantly, water-based electrodes are also easier to use than gel-based electrodes [6,7]. To evaluate the usability of this setup, the System Usability Scale (SUS) was employed [8]. Our primary developed VEP-EEG setup was evaluated on usability and user-friendliness in a previous study and was found highly user-friendly [9]. This study builds on that previous work, by testing the usability and user-friendliness of a second improved setup, with most importantly the addition of direct feedback for participants on electrode connection, in a population that is more representative of migraine patients than the population that evaluated setup 1. Migraine patients are not fundamentally different in how they would be able to operate both setups compared to the general population; however, older participants with a higher variety in educational background were included in the group that evaluated setup 2. This was done, since migraine primarily affects people during the working period of their life, and the group that evaluated setup 1 primarily consisted of young and highly educated participants.

Finally, the evaluation of setup 1 is added alongside that of setup 2 here to show the progress that was made in improving the setup and to compare the two.

The aim of this study was therefore to evaluate the usability and user-friendliness of an improved VEP-EEG telemonitoring setup for home-based research in a population that is more representative of migraine patients, using SUS score as the primary outcome measure, and assessing completion time, electrode connectivity, and participant experience as secondary outcomes. Additionally, the new and improved setup was compared to the first one.

## Methods

### Study design

Two separate groups of participants evaluated the 2 EEG setups. Group 1 evaluated setup 1, and group 2 evaluated setup 2. Setup 2 was improved based on the findings from the evaluation of setup 1 and was tested on usability by a different (older and more variety in educational background) population that was more representative of migraine patients than the first group. The most important improvement that was added to setup 2 is a direct feedback system, which informs participants if electrodes are sufficiently connected. Such a system was lacking in setup 1. Each participant first received an instruction guide and a demonstration explaining how to perform the EEG measurements. Participants then conducted the measurements independently on 2 separate occasions, with the interval between measurement days varying. After the first measurement day, they completed questionnaires assessing usability via the SUS score [8] and their overall experiences, and data was collected regarding completion time, and number of electrodes successfully connected. This was repeated after the second measurement day for overall experience, successful electrode connection, and completion time to identify a possible improvement over time. Feedback from participants was asked on both measurement days through open-ended questions.

The study was approved by the Medical Ethics Committee of Leiden University Medical Center (P24.025). Written informed consent was obtained from all participants. Data were collected between November 2024 and April 2025.

### Participants

Participants were aged ≥18 years, were proficient in Dutch, and were not required to have a migraine diagnosis, as the focus of the study was on evaluating the usability of the setup rather than analyzing VEP-EEG data. This meant that participants were a mixture of healthy volunteers and a few migraine patients. All migraine patients performed the measurements on interictal days, so no headache or other accompanying migraine symptoms occurred during the measurements. Exclusion criteria included (photosensitive) epilepsy and prior experience with EEG recordings. For some participants who did have migraine, migraine diagnoses were confirmed by a headache researcher and classified according to the International Classification of Headache Disorders (ICHD-3) [10].

### Outcomes

The primary end point was the comparison of SUS scores for setup 1 and setup 2 against the benchmark SUS score of 68, which is interpreted as indicating above-average usability [11]. The SUS questionnaire consisted of 10 items and was scored using the formula (*X* + *Y*) × 2.5, where *X* was the sum of the scores for all odd-numbered questions minus 5 and *Y* was 25 minus the sum of the scores for all even-numbered questions. In addition, potential predictors associated with higher SUS scores were explored. Secondary outcomes included completion time, number of electrodes successfully connected, and participants’ overall experience with the 2 setups. The completion time measurement began when participants started reading the instruction guide and ended when the measurement was completed. The successful electrode connectivity, out of 7 in total, was chosen as a task metric because of its simple user application. Moreover, the connectivity of the 3 occipital electrodes was determined separately, as they are deemed most important for the subsequent project, which aims to determine cortical excitability changes in the preictal phase of migraine attacks.

### Setup 1

An overview of the complete setup is shown in Fig. 1A. The setup included a wearable EEG-measuring device (APEX) with a water-based Infinity head cap (TMSi — an Artinis Company, The Netherlands). Seven electrodes of the headcap were used: 1 frontal electrode (Fz), 3 central electrodes (C3, Cz, and C4) and 3 occipital electrodes (O1, Oz, and O2). The electrodes in the cap were connected to the scalp via sponges, which were wet in salt water, and placed in the cap. The ground electrode was placed on the wrist by a wristband. APEX software was used to allow the researchers to view a feedback system indicating which electrodes were successfully connected. This feedback system was not visible for the participants. A study phone was added to the setup to allow participants to see if they placed the cap correctly on their head. The study phone had an application that functioned as a mirror for patients with straight lines that the app superimposed on their heads, which aided them in placing the cap straight and correctly on their head. This application could not provide direct feedback on electrode connection and could not show step-by-step visual instructions. It only functioned to aid in cap alignment. To be able to capture the responses to VEP stimulation, a control device and light-emitting diode (LED) goggles were used. The control device controlled the LED goggles to provide light flashes intermittently when required and played auditory cues to indicate different phases of the measurement protocol. Finally, participants received an extensive instruction guide on paper on how to perform the measurement independently.

**Fig. 1.**
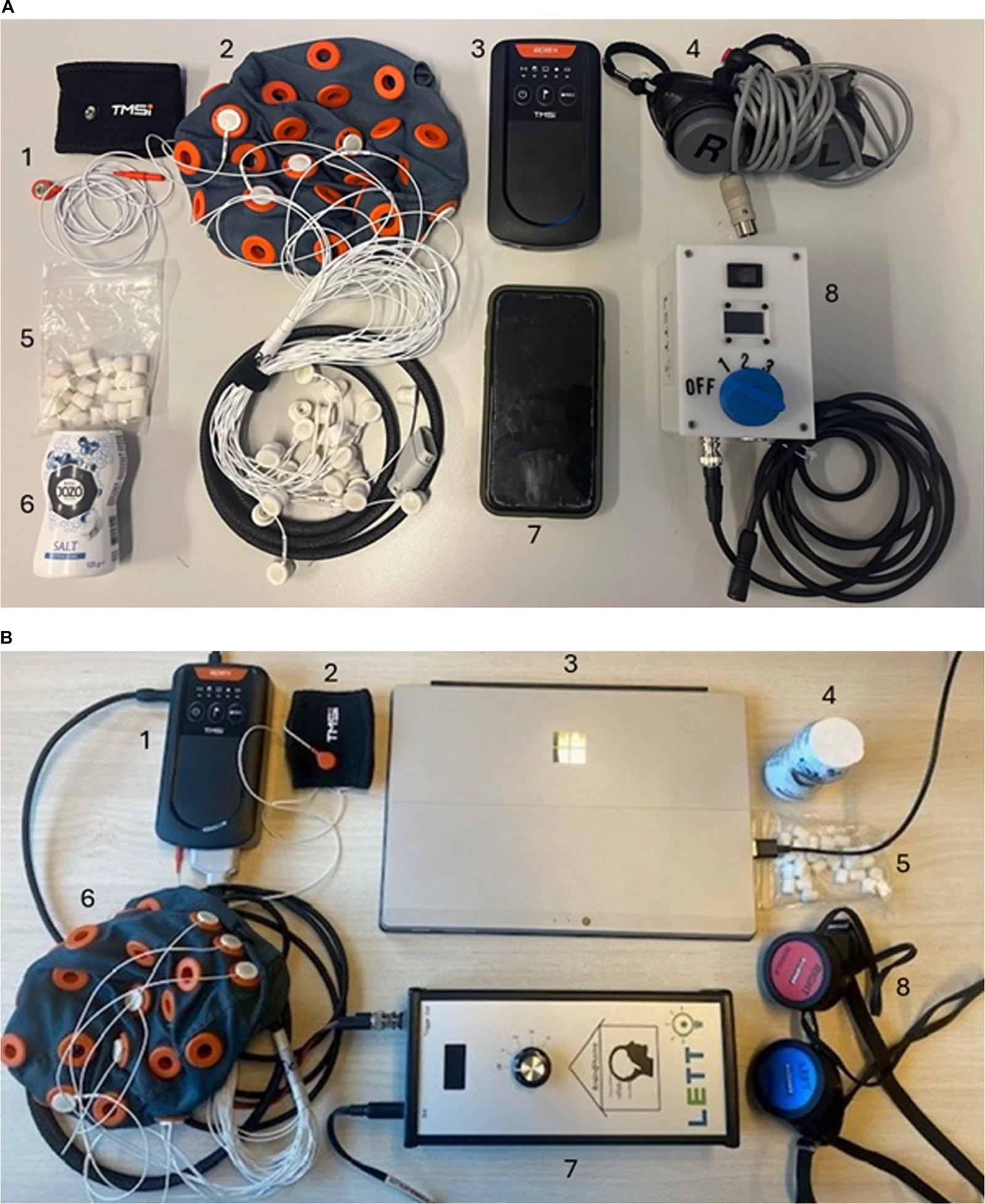
(A) Overview electroencephalogram (EEG) setup 1. 1 = Ground electrode with wristband. 2 = Water-based head cap with 7 electrodes. 3 = APEX, wearable EEG-measuring device. 4 = Light-emitting diode (LED) goggles. 5 = Sponges, to establish a connection between the electrodes in the cap and the scalp. 6 = Salt, to be added to the water in which the sponges were wet. 7 = Study phone, which served as a mirror for cap alignment. 8 = Control device, to control the LED goggles to provide the light stimulus (“Chirp”) protocol. (B) Overview EEG setup 2. 1 = APEX, wearable EEG-measuring device. 2 = Ground electrode with wristband. 3 = Tablet, which contains the Neurocenter software program. 4 = Salt, to be added to the water in which the sponges were wet. 5 = Sponges, to establish a connection between the electrodes in the cap and the scalp. 6 = Water-based head cap with 7 electrodes. 7 = Control device, to control the LED goggles to provide the light stimulus (“Chirp”) protocol. 8 = LED goggles.

Per measurement day, 2 types of measurements were conducted: the VEP-EEG protocol and the Resting State protocol. For the Resting State protocol, participants alternated between opening and closing their eyes every minute for a total of 5 min (cued by the tones played through the control device). For the VEP-EEG protocol, participants put on the LED goggles, through which light flashes consisting of a brief sequence (~6 s) with a rapidly increasing stimulation frequency from 10 to 40 Hz were emitted (4 pulses at each frequency). This stimulation protocol was repeated 12 times with a 10- to 15-s break while participants had their eyes closed. This protocol, which measures the brain’s response to a visual flash stimulus (quantified by the VEP), is called the “Chirp” protocol [12] and was validated during an earlier study [4]. The LED goggles, through which the actual visual stimuli will be given, are manufactured by Medelec and MicroMed with a light intensity of 2.64 log cd/m^2^ (438 lux) at wavelength 654 nm.

### Setup 2

An overview of the complete second setup is shown in Fig. 1B. The improvements made to setup 2 were based on feedback from participants who evaluated the first setup and were based on design improvement suggestions made by the researchers. The equipment required for setup 2 was somewhat similar to that of setup 1, with the important distinction of the addition of a direct feedback system on electrode connection for participants. This direct feedback system was made possible through the Neurocenter software program (Clinical Science Systems, Leiden, The Netherlands), which was running on an added tablet. To determine which electrodes were successfully connected, the direct feedback system used a flat-line detector and a schematic overview of the 7 electrodes and their positions on the head that turned green upon sufficient connection and otherwise stayed red. Participants were then instructed to keep pressing on the different electrodes until all turned green. The Neurocenter program also showed participants a step-by-step visual instruction (in addition to the paper instruction guide from setup 1) with voice-over guidance, aided in remote supervision as it allows remote live viewing of the measurements, and improved data acquisition and storage in an encrypted cloud-based facility.

The study phone was replaced with a mirror function in the Neurocenter program, and the control device was modified to be rechargeable and more durable. Finally, the Resting State protocol was extended from 5 to 10 min, as this duration was deemed necessary for the subsequent project, which aims to determine cortical excitability changes in the preictal phase of migraine attacks (ongoing Migraine@Home project). The “Chirp” protocol remained the same.

### Statistical analysis

Data cleaning and analysis were conducted using R statistical software (R version 4.2.1; R Foundation for Statistical Computing, Vienna Austria, 2016; https://www.R-project.org/). The sample size was determined based on a somewhat comparable study of at-home EEG monitoring for people with epilepsy [5].

For baseline characteristics, descriptive statistics were used and summarized as medians with interquartile ranges (IQRs) or frequencies with proportions. All participants were included in the analysis.

For the primary outcome, Wilcoxon signed-rank tests were performed to compare the SUS scores of group 1 and group 2 to the benchmark SUS score of 68. For crude comparison between group 1 and 2, a Mann–Whitney U test was used. Additionally, linear regression with adjustment for age, sex, educational level, and confidence in technical capabilities was used to compare the SUS score of group 1 to the SUS score of group 2.

Medians and means were used to describe the secondary outcomes: completion time, number of successfully connected electrodes, and overall experience for both groups. Additionally, to identify possible improvement over time, the difference between the first and second measurement days was evaluated within-group with a linear mixed-effects model for completion time, a generalized linear mixed model for electrode connection, and a cumulative link mixed model for overall experience, with time in-between sessions as a covariate. For skewed data, a log₂ transformation was applied.

Finally, between-group analyses were performed while accounting for repeated measurements. A comparison in improvement over time was made from the first to the second measurement day between groups using the interaction term between group and measurement day in a linear mixed model for completion time, in a generalized linear mixed model for electrode connection, and in a cumulative link mixed model for overall experience—once in a crude analysis, and once in an adjusted analysis where correction for time in-between sessions, age, sex, educational level and confidence in technical capabilities took place.

## Results

### Study population

A total of 40 participants were included, each group comprising 20 different participants with group 1 evaluating setup 1 and group 2 evaluating setup 2. All participants completed both measurement days and the associated questionnaires; no dropouts occurred. The mean interval was 6.7 d for group 1 and 5.3 d for group 2 between measurement days 1 and 2.

### Baseline characteristics

Out of 40 participants, 7 had a verified migraine diagnosis. The majority of participants in both groups were female (65% in group 1 and 75% in group 2). The median age was 26 years [IQR 22 to 48] in group 1 and 56 years [IQR 42 to 60] in group 2. All participants with migraine performed the measurements during the interictal phase. Additional baseline characteristics are presented in Table 1.

**Table 1.** Baseline characteristics

Characteristics	Participants (total)	Participants (setup 1)	Participants (setup 2)	*P* value
Number of participants, *n*	40	20	20	
Years of age, median (IQR)	27 [22, 48]	56 [42, 61]	0.001
Female sex, *n* (%)	28 (70%)	13 (65%)	15 (75%)	0.73
Migraine diagnosis, *n* (%)	7 (17.5%)	4 (20%)	3 (15%)	1.00
Highest educational level, *n* (%)				0.001
Primary education	1	1	0	
VMBO ^a^	0	0	0	
MBO ^b^	4	0	4	
HAVO/VWO ^c^	11	9	2	
HBO ^d^	14	5	9	
WO ^e^	10	5	5	
Confidence in technical capabilities, *n* (%)				<0.001
None	2 (5%)	1 (5%)	1 (5%)	
Somewhat	26 (65%)	19 (95%)	7 (35%)	
High	12 (30%)	0 (0%)	12 (60%)	

IQR, interquartile range

^a^ Prevocational secondary education

^b^ Secondary vocational education

^c^ Senior general secondary education/preuniversity education

^d^ Higher vocational education

^e^ Scientific/academic education

### SUS score

Group 1, consisting of the participants who evaluated setup 1, gave that setup a median SUS score of 91.2 [IQR 86.2 to 95.0] (Fig. 2). Group 2, consisting of the participants who evaluated setup 2, gave that second setup a median SUS score of 81.2 [IQR 70.6 to 90.6] (Fig. 2). Both scores exceeded the SUS benchmark of 68.0 (group 1: *P* < 0.001; group 2: *P* = 0.003), indicating that both setups were considered more than sufficiently user-friendly. SUS scores were only determined on measurement day 1 for both groups. Direct comparison of the evaluations of group 1 and group 2 resulted in a median difference in SUS scores of 7.5, confidence interval (CI): 0.0 to 17.5, *P* = 0.024 for crude analysis. However, that difference disappeared in the adjusted analysis; β = −9.9 for group 2, CI: −21.5 to 1.77, *P* = 0.094.

**Fig. 2.**
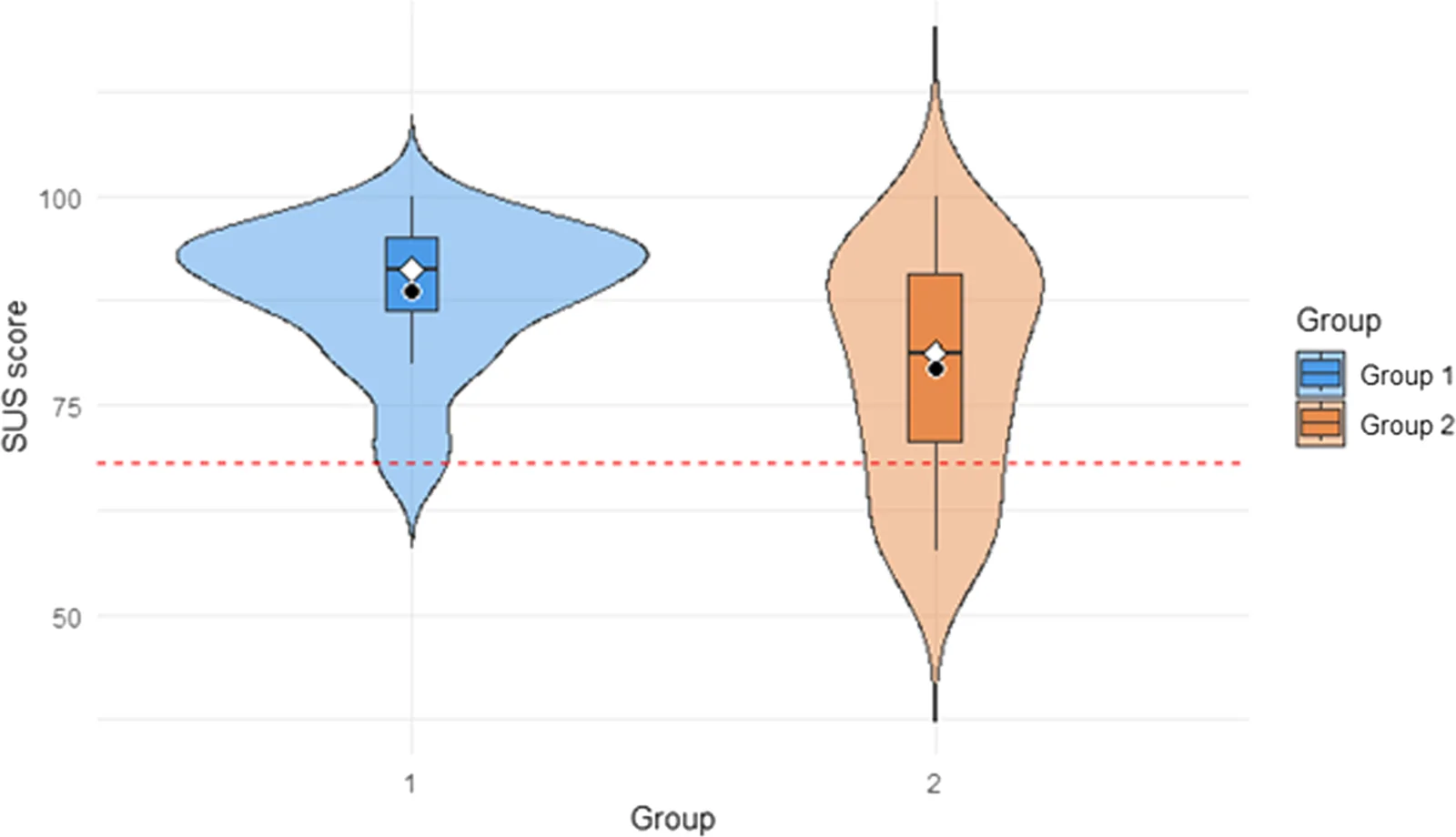
System Usability Scale (SUS)-score distribution per group compared to benchmark (68).

### Completion time

The distribution of completion times in 5-min intervals is shown in Fig. 3A and B for groups 1 and 2. In both groups, most participants completed the measurements within 20 to 25 min while keeping in mind that the Resting State protocol was extended from 5 to 10 min for group 2. Mean completion time in group 1 was 25.1 ± 0.66 min [95% CI: 23.7 to 26.5] on measurement day 1 and 21.8 ± 0.66 min [95% CI: 20.4 to 23.2] on measurement day 2. There was a significant reduction in completion time between the first and second measurement (−3.3 min, CI: −4.78 to −1.82, *P* < 0.001). In group 2, the mean completion time for the first measurement was 23.6 ± 0.66 min [95% CI: 22.3 to 25.0] and 23.9 ± 0.67 min [95% CI: 22.6 to 25.3] for the second measurement. Completion time in group 2 did not differ significantly between the 2 measurements (+0.4 min, CI: −1.11 to 2.00, *P* = 0.57). For the between-group analysis, the interaction between group and time was significant in both the crude (β = 3.74, 95% CI 1.67 to 5.82, *P* = 0.001) and adjusted models (β = 3.74, 95% CI 1.67 to 5.82, *P* = 0.001), indicating that the improvement over time differed significantly between groups with no improvement for group 2 and −3.3 min for group 1. Individual improvement over time for groups 1 and 2 separately is shown in Fig. 3C.

**Fig. 3.**
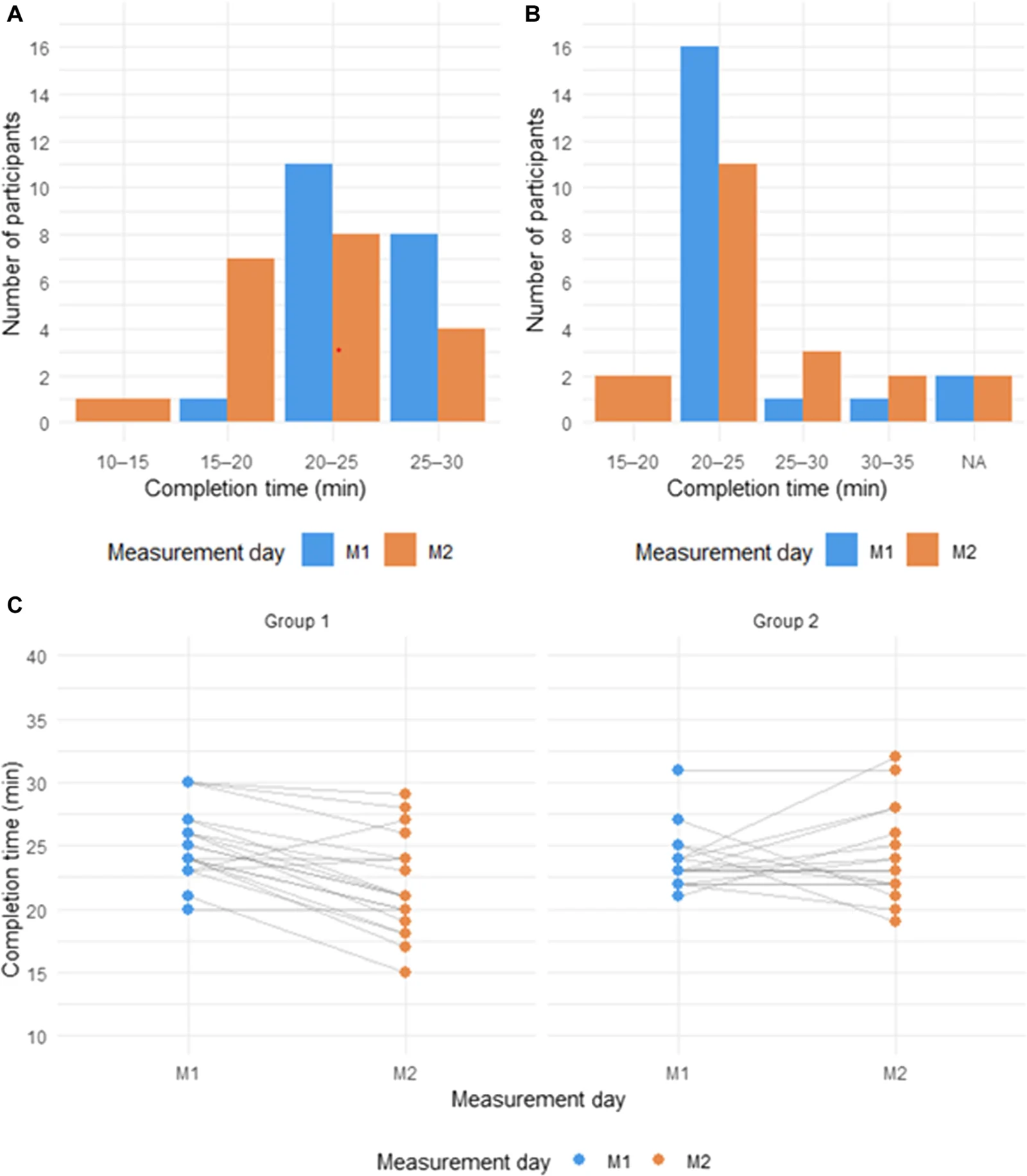
Completion time distribution for group 1 (A) and group 2 (B). Data on 2 participants are missing in group 2 (B). (C) Completion time improvement for group 1 and group 2. Data on 2 participants are missing in group 2.

### Electrode connection

The median number of successfully connected electrodes was 6 out of 7 for both group 1 [IQR 3.8 to 7.0] and group 2 [IQR 4.0 to 7.0], as shown in Fig. 4A and B. In group 1, a mean of 85.0% ± 22.0 of electrodes were connected at measurement day 1 compared to 75.7% ± 26.3 at measurement day 2, with an odds ratio (OR) of 2.1 [95% CI 1.1 to 4.0], *P* = 0.03. In group 2, mean electrode connection was similar at both measurement days (77.9% ± 34.2 vs. 75.0% ± 26.6), with an OR of 1.3 [95% CI 0.6 to 2.7], *P* = 0.47. For the 3 occipital electrodes specifically (O1, Oz, and O2), the mean percentage of successfully connected electrodes for group 1 at measurement day 1 was 85.0% ± 27.5 and 68.3% ± 39.7 at measurement day 2, with an OR of 4.2 [95% CI 1.2 to 15.2], *P* = 0.03. For group 2, the mean percentage of successfully occipital connected electrodes was 80.0% ± 38.1 at measurement day 1 and 71.7% ± 42.3 at measurement day 2, with an OR of 2.9 [95% CI 0.8 to 10.9], *P* = 0.12.

**Fig. 4.**
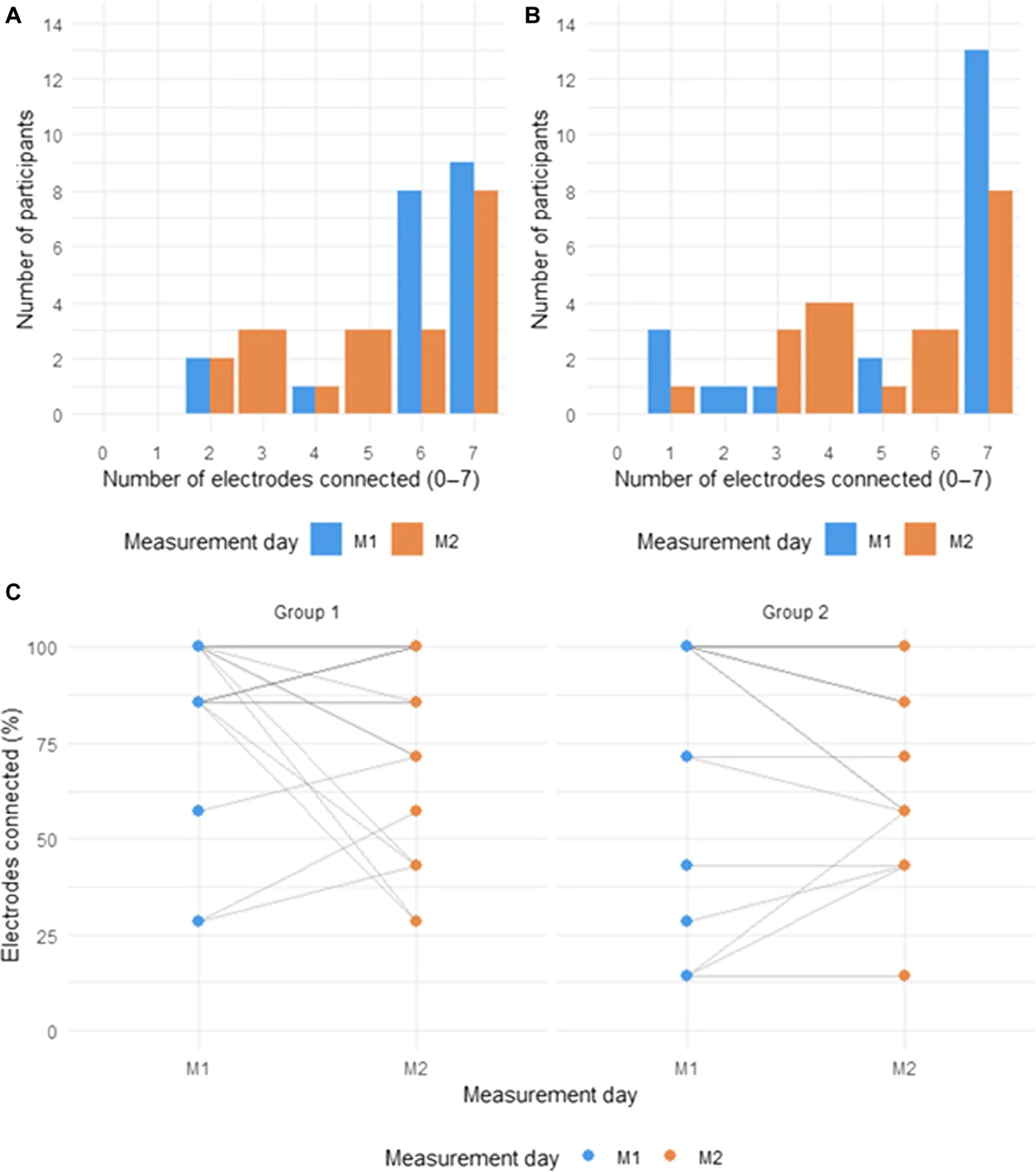
Number of electrodes (out of 7) successfully connected for group 1 (A) and group 2 (B). (C) Electrode connection improvement for group 1 and group 2.

For the between-group analysis, the interaction between group and time was not significant in the crude analysis (OR 1.67, CI: 0.63 to 4.43, *P* = 0.30) nor in the adjusted analysis (OR 1.69, CI: 0.64 to 4.46, *P* = 0.29), indicating that the improvement over time in electrode connection did not differ between the groups. However, in the adjusted analysis, female sex was a significant predictor for less electrode connection compared to male sex (OR 0.10, CI: 0.03 to 0.39, *P* = 0.0008). Individual improvement over time for groups 1 and 2 separately is shown in Fig. 4C.

### Overall experience

Both in group 1 and group 2, participants rated their overall experience with the setups as good to excellent with an overall median grade of 4/5 on both measurement days [IQR 4 to 5 for group 1, IQR 4 to 4 for group 2] (Fig. 5A and B). No significant change in overall experience was observed in group 1 from measurement day 1 to measurement day 2 (OR 1.51, CI: 0.35 to 6.52, *P* = 0.58). Group 2 did not differ in overall experience enough between those 2 measurement days to test a difference (Fig. 5B). Therefore, comparison between the groups in improvement over time was considered not meaningful.

**Fig. 5.**
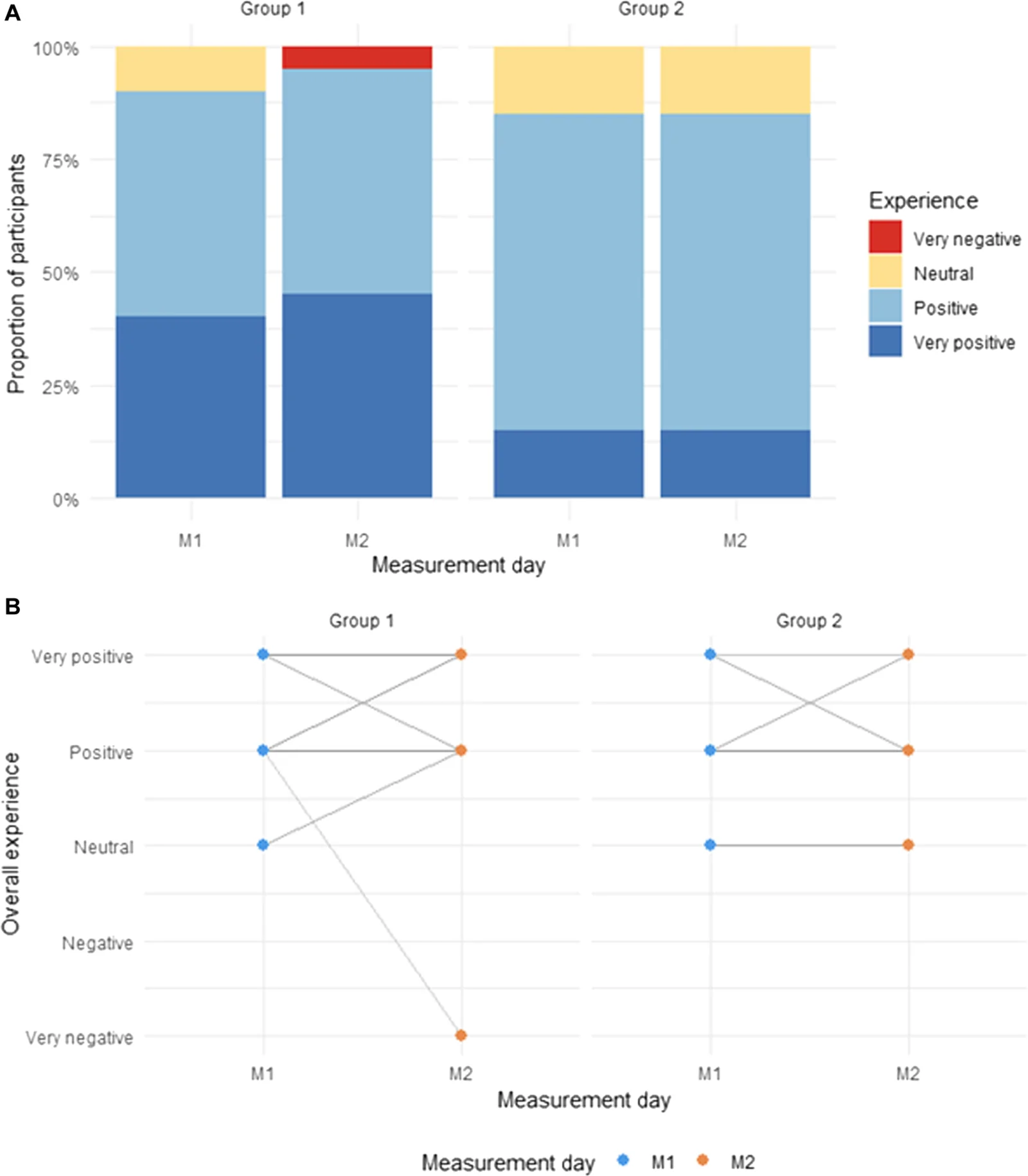
(A) Overall experience ratings for group 1 and group 2. (B) Overall experience improvement for group 1 and group 2.

### Feedback questions

Participants provided feedback following the measurements, through open-ended questions. For setup 1, positive comments frequently mentioned the instructional photographs in the paper instruction guide; however, the guide was deemed quite long, which did not help readability. The main challenge reported was determining adequate contact between the scalp and water-based electrodes, as a visual feedback system for participants was lacking. That is why the direct feedback on electrode connection was added to the second setup. Moreover, the Neurocenter software program was added on a tablet for the second setup, as this provided not only the direct feedback system but also a step-by-step visual instruction for participants, with a more condensed version of the steps and also voice-over guidance. Finally, the Neurocenter program aided the researchers in remote supervision of the measurements and improved data acquisition and storage in an encrypted cloud-based facility. Also, different cap sizes were added. For setup 2, participants again appreciated the visual instructions, both in the guide and on the tablet, as well as the stepwise format and voice-over guidance. Nonetheless, some difficulties with electrode connection remained, despite the addition of a visual direct feedback system. An extensive overview table of the feedback that participants submitted is shown in Supplemental File 1.

## Discussion

Two VEP-EEG telemonitoring setups for home-based research purposes were evaluated. Both setups demonstrated high usability, indicated by a median SUS score of 91.2 for setup 1 and a median SUS score of 81.2 for setup 2, exceeding the benchmark SUS score of 68, which is considered average. Comparison between the group that evaluated setup 1 and the group that evaluated setup 2 showed an initially significant difference in SUS score; however, this difference disappeared when corrected for age, sex, education level, and confidence in technical skills. The aim of this study was to show that usability and user-friendliness of an improved VEP-EEG setup (setup 2) remained sufficient in an older and more varied in educational background population sample. This is shown by the median SUS score of 81.2 for setup 2, since SUS scores from 80 and up are considered above average [13]. The evaluation of setup 1 was included to show the progress that was made in developing such a setup.

Additionally, completion time was evaluated, which showed a significant difference in improvement over time between the 2 groups. Group 1 was able to perform the measurement 3.3 min faster on measurement day 2 compared to day 1. For group 2, there was no improvement over time. This is thought to be due to the visual step-by-step instruction with voice-over guidance and direct feedback system on electrode connection in setup 2, which makes it easier for participants to follow the steps more efficiently directly on the first measurement day. In contrast, group 1 had a less clear instruction and no direct feedback system, which is why they may have benefitted from repetition more as is demonstrated by the shorter completion time on the second measurement day.

Next, the percentage of successfully connected electrodes decreased for group 1 from 85.0% on day 1 to 75.7% on day 2, while this remained more stable for group 2 (77.9% to 75.0%). This decrease over time was not significant between the groups; however, female sex was a significant predictor for less electrode connection. The decrease in group 1 can be explained by the fact that participants did not have a direct feedback system, so they did not know when they had pressed the electrodes sufficiently on the scalp. The varying percentage of successful electrode connection over time highlights the fact that establishing a sufficient electrode connection remains one of the most challenging aspects of home-based VEP-EEG monitoring. Finally, the negative influence of female sex on electrode connection can be explained by the fact that women typically have more and thicker hair on their scalp, which can impede the connection between the electrodes and the scalp.

Finally, both groups rated their overall experience with the setups as good to excellent, and this effect did not diminish over time.

These findings support the implementation of the final setup in the subsequent project, which aims to determine cortical excitability changes in the preictal phase of migraine attacks (Migraine@Home project).

While previous studies have evaluated telehealth solutions with the SUS, they typically involved simpler tasks such as symptom tracking or vital sign monitoring [14]. In contrast, this study required participants to independently perform complex procedures like EEG recordings. A comparable study evaluated a wearable EEG system for daily home monitoring in 11 epilepsy patients [5]. Their average SUS score for the EEG system was 63.9 (range from 42.5 to 75), while the median SUS score of setup 2 from this study was 81.2. The epilepsy study explains the lower SUS score by mentioning that most participants faced technical or practical challenges with the EEG system during the study period of 6 months. This was not the case for this study, however; only 2 measurements per participant were performed here.

Moreover, prior research has emphasized the role of telemedicine as both an intervention and an outcome measure in migraine care, particularly through tools like electronic diaries and video consultations [15]. Building on that, this study incorporated feedback from participants to improve the setup without compromising usability. These findings support the feasibility of integrating more sophisticated telehealth tools into migraine research, provided they are thoroughly evaluated using validated instruments such as the SUS. In addition to using a validated usability tool, this study included task metrics such as completion time and electrode connection, combining both quantitative and qualitative methods. Repeated measurements allowed assessment of potential learning effects, and participant feedback from open-ended questions was used to iteratively improve the setup.

A potential limitation of this study was the limited variation in age, education level, and confidence in technical capabilities among participants of the first group. To address this, the second group was selected to differ significantly from the first in these baseline characteristics. Interestingly, despite greater variation in education level, this group more frequently rated themselves as highly skilled in technical abilities.

Another potential limitation concerns the possibility that the number of successfully connected electrodes could be zero if the ground electrode was not properly applied, highlighting the complexity of the setup. To address this, instructions emphasized (both verbally and within the guide) that pressing individual electrodes would not establish a connection if all were disconnected and that reapplying the ground electrode was necessary; this approach resolved the issue in most cases. This solution was feasible only in setup 2, which included a feedback system indicating which electrodes were successfully connected, whereas setup 1 lacked such a feature for the participants. In this study, we used 2 methods for determining electrode connection: impedance and the flat-line detector (detection of nonzero signal values). For the first setup, an impedance interface was visible for the researchers but not for the participants. However, we did not assess impedance values according to the best clinical practices outlined in another study [16]. Therefore, data quality may vary across recordings. For the second setup, participants used a direct feedback system with a flat-line detector. It is currently still being researched if the feedback of the flat-line detector leads to comparable data quality as the feedback of an impedance marker. Additionally, signal quality of the water-based electrode systems has been previously validated [6,7].

A final limitation might be the fact that no within-subject design was used when performing this study, where the same participants would have tested both setups. The rationale for using 2 independent cohorts instead is based on practical reasons. After evaluating setup 1, it was found that that group was not really representative of migraine patients, since they were mainly young and highly educated participants. Therefore, for setup 2, a different group was chosen with more variety in age and educational background, since migraine primarily affects people during their working years. Moreover, improvements to the setup were already implemented, so it was deemed not useful for the second group to evaluate setup 1 as well. In short, this study aimed to answer the question if the new and improved setup [2] is user-friendly enough in a population sample that is more representative of migraine patients. The results seem to confirm this hypothesis.

Validating the usability of self-administered EEG for research purposes enhances scalability and addresses logistical challenges compared to in-hospital recordings, while capturing data in a more natural environment. Improvements made from setup 1 to setup 2 enhanced both user-friendliness and data preservation. These enhancements support the implementation of the second and final setup in the main Migraine@Home project, facilitating reliable home-based EEG recordings with positive user experiences and improved quality data preservation.

This study has shown that direct feedback on electrode connection is of the utmost importance when conducting VEP-EEG home-based research. Furthermore, since the Neurocenter program also works for remote supervision, future research applications could focus on remote supervision in combination with guidance via telephone where necessary while participants perform the measurements independently at home. This study represents a first step toward migraine prediction using a home-based VEP-EEG setup with a focus on user-friendliness and practical application; however, further studies are required to validate those possible predictive EEG biomarkers. This will be the focus of the Migraine@Home project.

Telemonitoring using an improved home-based VEP-EEG setup with direct feedback on electrode connection is feasible. This improved setup demonstrates high user-friendliness in a population sample that is more representative of migraine patients.

## Ethical Approval

The study is performed in accordance with the Declaration of Helsinki Ethical Principles and Good Clinical Practices and was approved by the local ethics committee.

## Data Availability

The data that support the findings of this study are available from the principal investigator, G.M.T.
