# Supplementary material for: Feasibility and Usability of Home-Based Visual Evoked Potential Electroencephalogram in Neurological Telemonitoring Research
Source: Comput Struct Biotechnol J. 2026 May 15;35(2):0081. doi: 10.34133/csbj.0081 (PMC13632936; doi:10.34133/csbj.0081)
Supplement: Supplementary file 1 [file csbj.0081.f1.docx]

**Supplemental File 1.**

| **Feedback setup 1** | **Item** | **Mentioned by % of participants** |
| --- | --- | --- |
| Positive | Photographs in instruction guide | 50% |
| Positive | Red-highlighted key terms in instruction guide | 15% |
| Positive | Step-by-step structure | 15% |
| Positive | Verbal explanation before start procedure by researcher | 10% |
| Difficulty | Establishing successful electrode connection | 30% |
| Difficulty | Putting on LED goggles without displacing the cap | 20% |
| Difficulty | Fragility of some cables | 15% |
| Difficulty | Confusion with device names | 10% |
| Suggestion for improvement | Shortening the instruction guide | 15% |
| Suggestion for improvement | Including more photographs in instruction guide | 10% |
| Suggestion for improvement | Implementing different cap sizes | 5% |

| **Feedback setup 2** | **Item** | **Mentioned by % of participants** |
| --- | --- | --- |
| Positive | Photographs in instruction on tablet | 40% |
| Positive | Voice-over guidance on tablet | 30% |
| Positive | Step-by-step structure | 35% |
| Positive | Verbal explanation before start procedure by researcher | 25% |
| Positive | Availability of both condensed instruction on tablet and extensive paper instruction guide | 15% |
| Difficulty | Establishing successful electrode connection | 40% |
| Difficulty | Slow response time of tablet | 25% |
| Difficulty | Identification of front and back of cap | 10% |
| Difficulty | Putting on LED goggles without displacing the cap | 30% |
| Suggestion for improvement | Shortening the instruction even further | 20% |
| Suggestion for improvement | Removing of unnecessary wires | 5% |
| Suggestion for improvement | Expansion of the voice-over guidance | 30% |
